# Supplementary material for: Development of Olive Oil Containing Phytosomal Nanocomplex for Improving Skin Delivery of Quercetin: Formulation Design Optimization, In Vitro and Ex Vivo Appraisals
Source: Pharmaceutics. 2023 Mar 31;15(4):1124. doi: 10.3390/pharmaceutics15041124 (PMC10145320; doi:10.3390/pharmaceutics15041124)
Supplement: Supplementary file 1 [file pharmaceutics-15-01124-s001.zip › pharmaceutics-2273600-supplementary.pdf]

Optimization of UHPLC conditions

The effective determination of QC was achieved on ACCLAIM™ 120 C18 column and isocratic elution at flow rate of 0.7 mL/min with a mobile phase consisting of water acidified by phosphoric acid (pH ~3) and acetonitrile. A different proportion of water acidified by phosphoric acid (pH ~3) and acetonitrile was tested; (40: 60 v/v). Also the detection wavelength and column temperature were tested and 210 nm and 25 °C were chosen. Under the optimum condition; the total run time of the HPLC method was 7 min. QC was detected at 5.27 min as retention time as shown in Fig. S1. Calibration curve was linear over a range of 0.02-80 µg/mL (Fig. S2), and the equation was A=1.3633C +0.2755 with a correlation coefficient (r2) value of 0.9998 as depicted in Table S1.

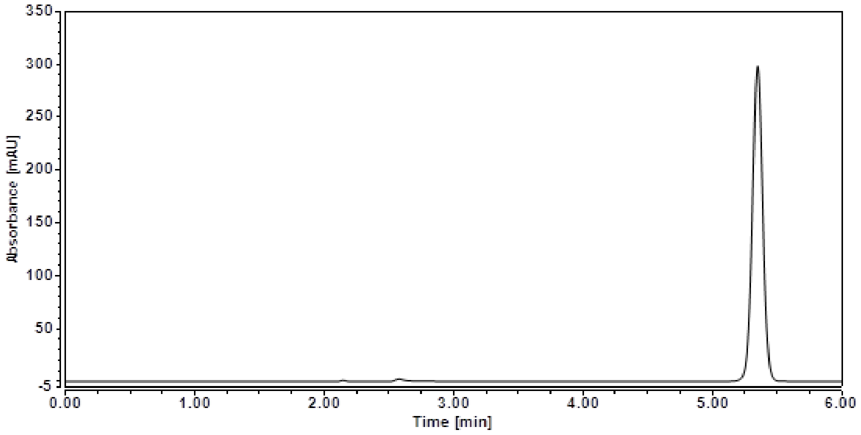

Figure S1. HPLC chromatogram of QC

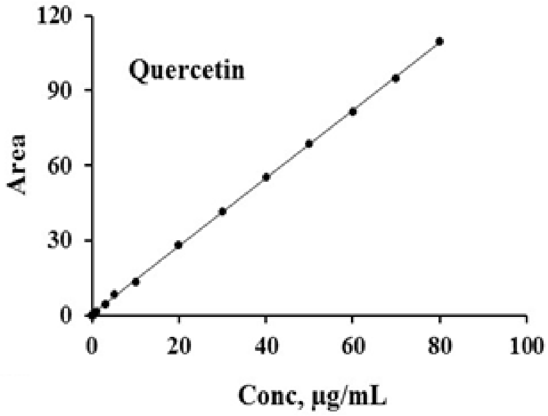

Figure S2: Calibration curve of QC

Table S1. Results of assay validation parameters of the proposed HPLC method for determination of QC

Parameter	QC
**Range**	0.02- 80 µg/ mL
**Slope**	1.3633
**Intercept**	0.2755
**R^2**	0.9998
**LOD***	0.004 µg/ mL
**LOQ***	0.0133 µg/ mL
**Recovery %**	94.41- 98.19
**RSD%**	0.66 - 2.80

Table S2: ANOVA for Quadratic model of response 1: Particle Size

Source	Sum of Squares	df	Mean Square	F-value	p-value	
**Model**	7761.69	9	862.41	20.72	0.0003	significant
A-Olive oil: PC ratio	915.92	1	915.92	22.01	0.0022	
B-PC:QC ratio	1367.64	1	1367.64	32.86	0.0007	
C-Concentration of surfactant	1716.98	1	1716.98	41.26	0.0004	
AB	2097.64	1	2097.64	50.40	0.0002	
AC	9.00	1	9.00	0.2163	0.6560	
BC	59.29	1	59.29	1.42	0.2715	
A^2	739.21	1	739.21	17.76	0.0040	
B^2	750.41	1	750.41	18.03	0.0038	
C^2	60.80	1	60.80	1.46	0.2660	
**Residual**	291.32	7	41.62			
Lack of Fit	204.20	3	68.07	3.13	0.1499	not significant
Pure Error	87.12	4	21.78			
**Cor Total**	8053.02	16				

Table S3: ANOVA for Quadratic model of response 2: Zeta potential

Source	Sum of Squares	df	Mean Square	F-value	p-value	
**Model**	81.37	9	9.04	4.70	0.0268	significant
A-Olive oil: PC ratio	12.00	1	12.00	6.24	0.0412	
B-PC:QC ratio	13.26	1	13.26	6.89	0.0342	
C-Concentration of surfactant	1.71	1	1.71	0.8890	0.3771	
AB	20.70	1	20.70	10.75	0.0135	
AC	14.82	1	14.82	7.70	0.0275	
BC	0.0900	1	0.0900	0.0468	0.8350	
A^2	8.91	1	8.91	4.63	0.0684	
B^2	1.62	1	1.62	0.8408	0.3897	
C^2	8.02	1	8.02	4.17	0.0806	
**Residual**	13.47	7	1.92			
Lack of Fit	10.40	3	3.47	4.51	0.0897	not significant
Pure Error	3.07	4	0.7680			
**Cor Total**	94.84	16				

Table **S4: ANOVA for Quadratic model of response 3: EE%**

Source	Sum of Squares	df	Mean Square	F-value	p-value	
**Model**	296.74	9	32.97	22.96	0.0002	significant
A-Olive oil: PC ratio	17.11	1	17.11	11.92	0.0107	
B-PC:QC ratio	21.45	1	21.45	14.94	0.0062	
C-Concentration of surfactant	118.58	1	118.58	82.59	< 0.0001	
AB	0.0225	1	0.0225	0.0157	0.9039	
AC	31.36	1	31.36	21.84	0.0023	
BC	0.8100	1	0.8100	0.5642	0.4771	
A^2	103.06	1	103.06	71.78	< 0.0001	
B^2	0.0400	1	0.0400	0.0279	0.8721	
C^2	6.34	1	6.34	4.42	0.0736	
**Residual**	10.05	7	1.44			
Lack of Fit	7.42	3	2.47	3.77	0.1164	not significant
Pure Error	2.63	4	0.6570			
**Cor Total**	306.79	16				

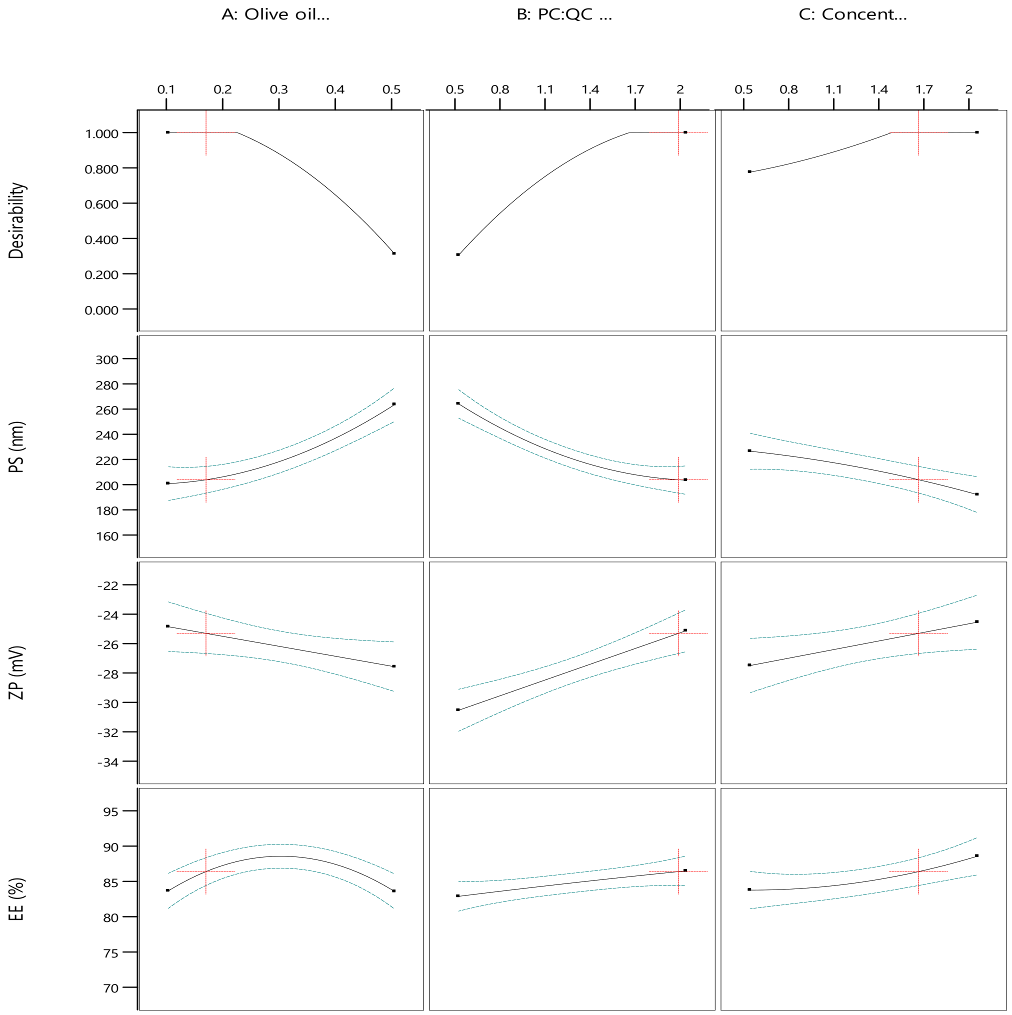

Figure S3: Predicted and actual values of independent variables and responses according to the desirability.
